# Institutional Notes and News

**Published:** 1911-07-22

**Authors:** 


### Institutional Notes and News.

**Effect of Insurance Bill on Norwich Hospital.**

The probable effect of the Insurance Bill was vigorously discussed at the recent quarterly meeting of the Norfolk and Norwich Hospital. Mr. R. E. Horsfall, after remarking that he hoped the question of the amalgamation with the Norfolk and Norwich Eye Infirmary would remain in abeyance till more was known of the terms and probable working of the National Insurance Bill, said that at a large meeting held in Birmingham of twenty-seven Midland hospitals (for a full report of which see The Hospital of June 24, page 325), it was resolved that if the Bill were passed in its present form it would affect their income and curtail their work. Another large meeting of the British Hospitals Associations was held recently in London (The Hospital of June 10, page 269), at which the Chairman of our Finance Committee was present. "A feature of the Bill which may affect hospitals is contained in Clause 12. If an insured person is an inmate of a hospital he will not receive any sickness or disablement benefit. At the discretion of the Friendly Society or of the Local Health Committee the money which he would otherwise receive may, in whole or in part, be applied for the relief of his dependants, and if he has no dependants the money shall be paid to the Local Health Committee towards its general purposes. Thus, under certain circumstances, it will be an actual gain to the insurance funds that the patient should be in hospital rather than be treated at home. In this way the hospitals whilst receiving no benefits whatever will actually be used as a means of increasing the insurance funds. I think, therefore, in view of the fact that we are unable to meet our own liabilities, it would be simply courting financial disaster to take over an institution whose last balance sheet showed an expenditure which exceeded the income by one-third. Much as the amalgamation would, undoubtedly, suit the convenience of the public and the medical profession, I maintain that the well-being of our own hospital ought to be our first consideration." Mr. C. Larking feared the hospital's debt will be further considerably increased. "It is many years," he said, "since we have sold any of our stock, and in being compelled to do so we are met with two grave difficulties; one is, that we should have to sell that which cost the Hospital considerably more; and the other that our income would be immediately reduced by about £200 per year. For 140 years it has been one of the traditions of this hospital 'to construct and not to destroy,' but I am expressing my personal opinion in saying that the legislation of the present day is nothing more or less than 'destruction and not construction.' The National Insurance Bill encroaches upon the activities of our institutions, overlaps with the best work of our public health authorities and trade unions, and places practical men and experienced associations under the dominance of a new army of official novices. A few days since I paid a visit to one of the largest hospitals in the West of England, and the secretary there informed me that although his hospital has received £43,000 towards an appeal of £45,000, they are considering whether they are justified in going on with their building scheme until they know how this new Bill will affect them. If the means of obtaining voluntary support be cut away from us the only alternative is State aid or municipalisation of hospitals. Now, a municipal body is not the right kind of authority to manage hospitals. They should be managed by people attracted to the work by their intense desire to do something for their fellow creatures, and not by persons who have been elected for some entirely different purpose. Again, voluntary hospitals, as you are aware, stand considerably higher than Poor Law Institutions, for the simple reason that most of their administration and work is done by voluntary workers, and, another thing, the nursing staff is drawn from a different status of women than would be otherwise the case if they were engaged by the State or municipal authorities." Mr. W. B. Greenfield thought it would be of advantage if the citizens had an opportunity of reading a full report of the remarks of Mr. Larking. It was something new to him that the Bill was going to have such an effect as Mr. Larking had predicted. As a trade-unionist and as a Friendly Society member, he did not think that there was any objection to Mr. Lloyd George's scheme.

**The Women's Hospital at Clapham.**

No maternal death in 549 confinements is a fact worth noting in the annual report of the Clapham Maternity Hospital. The public again may be reminded that the hospital was opened in 1889 to enable women to be attended in their confinements by doctors of their own sex. Besides this, an attempt has been made to provide hospital accommodation for the better class of unmarried girls from rescue homes, and the means have been more or less provided by which women may be trained in midwifery by women doctors. The secretary of the hospital is also a woman, Miss Marion Ritchie, we understand, holding that office with the post of treasurer. The hospital has fifty beds, and patients are received upon a sliding scale of payment from half-a-guinea to thirty shillings per week.

**Metropolitan Hospital and Insurance Bill.**

In presiding at the festival dinner of the Metropolitan Hospital, in Kingsland Road, the Hon. Alfred Lyttelton reminded the company that last year nearly 50,000 out-patients had been received. "This institution," he said, "is quite unique among the London hospitals in its provision of provident dispensaries. I believe in this matter those who have managed the hospital have shown a rare prescience of what is likely to occur, for I believe that the policy which had been pursued was the policy which will adapt itself to the changed conditions that we must shortly expect." Mr. Lyttelton supported the view that the out-patient department should be a consulting centre, to which no patient should go till a general practitioner had certified him as eligible. Mr. Leopold de Rothschild, the treasurer, thought the hospitals should combine together to elaborate a scheme. Mr. G. S. Buchanan, the secretary, announced subscriptions amounting to £5,880.

**Year's Work at Drumcondra Hospital.**

Colonel Gore Lindsay presided over the annual meeting of the Drumcondra Hospital, Dublin, last week. He congratulated the meeting on the results for the past year, in spite of the deficit. The report was read by the Rev. Fergus W. Greer, who gave the in-patients as 279, 253 of whom were paying patients. The Dublin Corporation has increased its annual grant to £62 10s. During the year the debt had been reduced from £163 to £152. Mr. Greer reminded the meeting that the Drumcondra Hospital kept its wards open all the year round. His Honour Judge Orr, in seconding, pointed out that if it had not been for the late Mr. Malone's gift of £190, the hospital would have been nearly £300 to the bad. On the motion of Mr. W. J. Arnold, seconded by Sir John Moore, the following were elected to serve on the Committee for the ensuing year: The Rev. P. J. M'Grath, C.C.; Mr. Charles Allen, V.S.; Mr. Richard Jones, J.P.; Mr. James Robertson, J.P.; and Mr. J. J. Simmington.

